# Infective Endocarditis by *Yersinia* Species: A Systematic Review

**DOI:** 10.3390/tropicalmed6010019

**Published:** 2021-02-02

**Authors:** Petros Ioannou, Georgios Vougiouklakis, Stella Baliou, Eugenia Miliara, Diamantis P. Kofteridis

**Affiliations:** 1 Department of Internal Medicine & Infectious Diseases, University Hospital of Heraklion, Heraklion, 71110 Crete, Greece; billahem@hotmail.com (G.V.); eugmiliara@gmail.com (E.M.); kofterid@uoc.gr (D.P.K.); 2 National Hellenic Research Foundation, 11635 Athens, Greece; stellabaliou@gmail.com

**Keywords:** endocarditis, systematic review, *Yersinia*, antimicrobial susceptibility

## Abstract

*Yersinia* spp. are non-spore-forming Gram-negative bacilli. They comprise only three species known to cause disease in humans, namely *Y. pestis*, *Y. enterocolitica* and *Y. pseudotuberculosis*. Since infective endocarditis (IE) is rarely caused by *Yersinia*, the management of these infections can be problematic due to the lack of experience. The purpose of this study was to systematically review all published cases of IE by *Yersinia* species in the literature. A systematic review of PubMed, Scopus and Cochrane Library (through 1 November 2020) for studies providing epidemiological, clinical and microbiological information as well as data on treatment and outcomes of IE caused by *Yersinia* species was performed. A total of 12 studies, containing data of 12 patients, were included. A prosthetic valve was present in 17% of patients. The mitral valve was the most commonly infected site, followed by the aortic valve. Fever, sepsis and embolic phenomena were common clinical signs, followed by heart failure. Aminoglycosides, cephalosporins and quinolones were the most commonly used antimicrobials. Clinical cure was noted in 83%, while overall mortality was 17%. This systematic review describes IE by *Yersinia* and provides information on patients’ epidemiology, clinical signs and the related therapeutic strategies and outcomes.

## 1. Introduction

*Yersinia* spp. are non-spore-forming Gram-negative bacilli of 1–3 μm in length and 0.5–0.8 μm in width. Apart from *Y. pestis*, all species are motile between temperatures of 22 and 30 °C and they are non-motile at 37 °C. *Yersinia* spp. are grown in aerobic and anaerobic cultures on non-selective and selective media, between 0 and 45 °C [1,2]. Initially, the *Yersinia* genus was discovered in 1894 by Alexander Yersin and Shibasaburo Kitasato and it currently consists of 18 species, while only three are known to cause disease in humans, namely *Y. pestis*, *Y. enterocolitica* and *Y. pseudotuberculosis*. Even though *Y. pestis* is known to cause plague and has caused the deaths of millions of people, *Y. enterocolitica* and *Y. pseudotuberculosis* are known to give rise to self-limited gastrointestinal diseases [1]. In particular, *Y. enterocolitica* consists of a heterogenous group of strains which are traditionally classified by biotyping into six biogroups, according to their phenotype, and into more than 57 O serogroups, based on their O surface antigen [2]. However, few of those serogroups, mainly serogroups O:3, O:5,27, O:8 and O:9, cause disease in humans [3]. Although gastroenteritis is the most frequent clinical manifestation of yersiniosis, other clinical manifestations may occur, including terminal ileitis, mesenteric adenitis, septicemia and reactive arthritis.

On the other hand, infective endocarditis (IE) is a rare disease that is associated with notable morbidity and mortality [4,5]. Even though it is very uncommon, IE by Gram-negative bacteria can be quite problematic due to inadequate experience and the paucity of data and guidelines regarding its treatment [5]. More specifically, IE by non-HACEK [Haemophilus, Aggregatibacter (previously Actinobacillus), Cardiobacterium, Eikenella, Kingella] Gram-negative bacteria is an entity that used to be associated with intravenous drug users for decades, even though in recent years, there are increasing reports of IE by these microorganisms associated with the healthcare system [6,7]. IE by non-HACEK Gram-negative bacteria is associated with a notable risk of mortality and morbidity, such as development of intracardiac abscess and vascular complications such as peripheral embolism or stroke [6,7]. The most commonly identified Gram-negative microorganisms associated with IE by Gram-negative bacteria in the literature are *Escherichia coli*, *Pseudomonas aeruginosa* and *Klebsiella* species; however, IE caused by other non-HACEK Gram-negative bacteria has been described in the literature [6,8,9]. This disease often requires a combination of antimicrobials along with surgery in order to achieve a cure [6]. However, depending on the species, there may be specific problems associated with its treatment, such as antimicrobial resistance [8].

Interestingly, even though there are some case reports with a literature review on IE by *Yersinia* species, a review adequately summarizing all available evidence on the topic is lacking [10].

The purpose of this study was to systematically review all cases of IE by *Yersinia* species in the literature and describe their epidemiology, microbiology, clinical signs, treatment and outcomes.

## 2. Materials and Methods

### 2.1. Data Search

In this review, the Meta-analysis of Observational Studies in Epidemiology (MOOSE) guidelines, were used [11]. Eligible studies were identified through search of the following databases: PubMed, Scopus and Cochrane Library, using the keywords: Yersinia AND endocarditis. The day of the last search was 1 November 2020.

### 2.2. Study Selection

Studies were included in the analysis if they met the following criteria: (1) published in English; (2) reported patients’ clinical characteristics, microbiology, treatment and outcomes. Studies meeting any of the following criteria were excluded from the analysis: (1) Secondary research papers (e.g., reviews), editorials and papers not associated with primary research; (2) studies focused on animal models and not on humans; (3) studies not written in English. Two independent investigators (P.I. and G.V.), using Abstrackr [12], independently reviewed the titles and abstracts of the resulting references and then retrieved and rescreened the full-text publications of potentially relevant articles. Study selection was based on consensus. Reference lists of included studies were searched for relevant articles. In the case where the investigators were unable to access a full-text publication, attempts were made to communicate with the study authors in order to kindly provide the full text.

### 2.3. Outcomes of Interest

The primary outcomes of the study were to record data on the (a) epidemiology of patients with IE by *Yersinia* species and (b) patients’ outcomes. Secondary outcomes were to record data on (a) the exact site of infection, (b) the patients’ clinical characteristics, (c) their antimicrobial susceptibility and (d) their treatment. The identification of independent risk factors for mortality was another endpoint of this study.

### 2.4. Data Extraction and Definitions

Data from each eligible study were extracted by two investigators (P.I. and G.V.). The extracted information included type of study, year of publication and country; patient demographic data (age and gender); patients’ relevant medical history (i.e., previous cardiac surgery or cardiac valve replacement and time after cardiac valve replacement); data related to infection data and microbiology of patients (including infection site, isolated strains, presence of complications and presence of embolism); treatment administered for IE; and outcomes (i.e., cure or death). Data on microbiology and relation of death to the index infection were reported according to the study authors. Diagnosis of IE was confirmed by the investigators based on information provided by the authors and the modified Dukes’ criteria if the diagnosis was definite (2 major or 1 major and at least 3 minor criteria, or 5 minor criteria) or if pathological data established a diagnosis of IE [13]. The complications recorded involved any organ dysfunction or clinical deterioration that was considered by the authors to be related to the IE. Sepsis was defined as the presence of two out of four of the following criteria: (a) temperature <36 °C or >38 °C; (b) heart rate >90 beats per minute; (c) respiratory rate >20 breaths per minute; (d) white blood cell count <4000/mm^3^, >12,000/mm^3^ or >10% immature (band) forms [14]. The quality of the evidence of the recorded studies’ outcomes was evaluated using the Grading of Recommendations Assessment, Development and Evaluation (GRADE) [15].

### 2.5. Statistical Analysis

Data are presented as number (%) for categorical variables and median (interquartile range, IQR) or mean (+/− standard deviation, SD) for continuous variables. A univariate logistic regression analysis was performed to identify factors associated with all-cause mortality of patients with IE by *Yersinia* species. The above-mentioned statistics were calculated using GraphPad Prism 6.0 (GraphPad Software, Inc., San Diego, CA, USA).

## 3. Results

### 3.1. Literature Search

**Figure 1:**
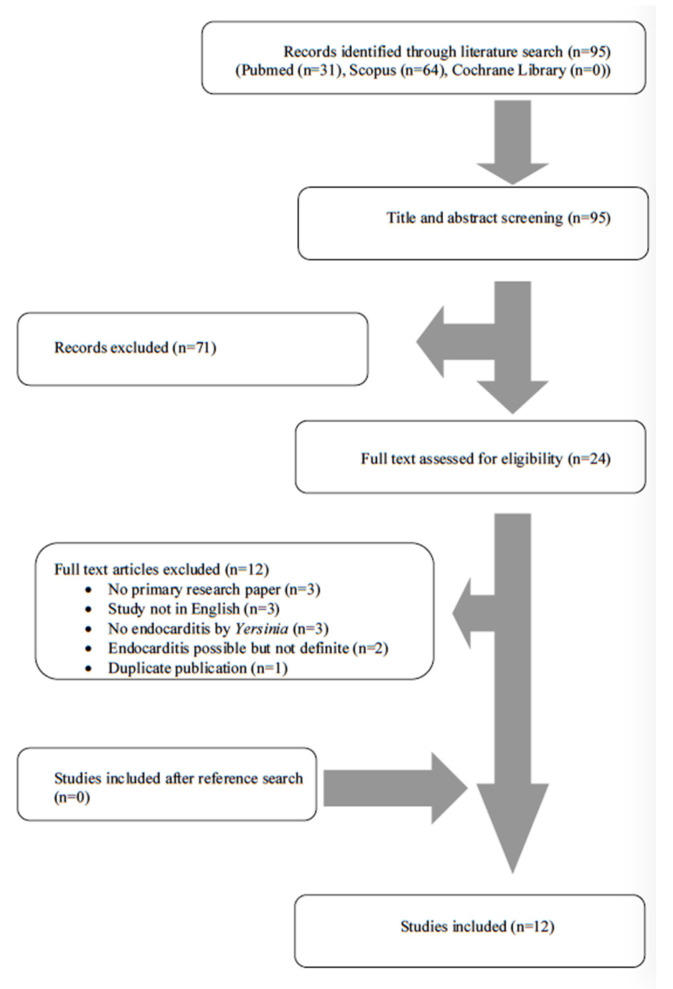
Flow diagram of study inclusion.

A total of 95 articles from PubMed, Scopus and Cochrane Library were screened. After reviewing the titles and abstracts, 24 articles were selected for full-text review. Of those studies, 12 were excluded from the review since three studies did not include original data (review articles), three studies were not written in English, three studies did not describe endocarditis by *Yersinia* species, two studies described cases with possible, but not definite, endocarditis and one study was a duplicate publication. Finally, 12 met the present study’s inclusion criteria [10,16,17,18,19,20,21,22,23,24,25,26]. The review process is graphically presented in Figure 1.

### 3.2. Included Studies’ Characteristics

The 12 studies that were finally included in the present analysis involved 12 patients in total. Supplementary Table S1 summarizes the characteristics of the included studies. Among those studies, 11 were conducted in Europe [10,16,18,19,20,21,22,23,24,25,26] and one in North America [17]. There were 12 case reports; thus, the overall quality of the evidence that contributed to this systematic review was rated as low to very low [15].

### 3.3. Epidemiology of IE by Yersinia Species

Age of patients ranged from 45 to 89 years, the mean age was 67 years [10,16,17,18,19,20,21,22,23,24,25,26] and 83% (10 out of 12 patients) were male [16,18,19,20,21,22,23,24,25,26]. A prosthetic cardiac valve was present in 17% (2 out of 12 patients) and was metallic in the mitral position in both patients [20,24], while 25% (three patients) had a history of rheumatic heart disease [16,20,24]. At the time of diagnosis of IE, 17% (two patients) had a permanent pacemaker [21,22]. Table 1 shows the characteristics of patients with IE, caused by *Yersinia*.

### 3.4. Microbiology and Antimicrobial Resistance of IE by Yersinia Species

The only identified species in all included patients was *Y. enterocolitica*. In particular, serotype O:3 was identified in 42% (five patients) [16,18,21,22,25] and serotype O:9 in 17% of cases (two patients) [20,24], whereas in 42% (five patients), the serotype was not identified [10,17,19,23,26]. A stool culture was positive in 17% (two patients) [16,22]. Resistance to aminopenicillins was noted in 71% (five out of seven patients) [10,18,19,21,26], to quinolones in 25% (two out of eight patients) [10,24], to chloramphenicol in 20% (one out of five patients) [24], to cephalosporins in 18% (two out of 11 patients) [17,24], to co-trimoxazole in 17% (one out of six patients) [24], to tetracycline in 17% (one out of six patients) [24], to carbapenems in 14% (one out of seven patients) [24] and to aminoglycosides in 8% of cases (one out of 12 patients) [24]. Table 1 illustrates the antimicrobial resistance to *Yersinia* species.

### 3.5. Diagnosis of IE by Yersinia

The most frequent site of infection was the mitral valve in 58% of cases (seven out of 12 patients) [16,18,20,21,23,24,26], followed by the aortic valve in 33% (four patients) [10,17,19,26] and the tricuspid valve in 17% (two patients) [22,25]. In 8% (one patient), multiple valves were infected [26]. In 8% (one patient), pacemaker infection was evident [22]. Diagnosis was facilitated with transthoracic echocardiography in 58% (seven out of 12 patients) [10,16,17,19,21,25,26] and transesophageal echocardiography in 25% of cases (four patients) [20,22,23,24], while diagnosis was set at autopsy in 8% (one out of 12 patients) [18].

### 3.6. Clinical Characteristics of IE by Yersinia Species

Fever was present in 92% (11 out of 12 patients) [10,16,17,18,19,20,21,22,23,24,25], sepsis in 70% (seven out of 10 patients with available data) [10,16,17,18,20,24,25] and embolic phenomena in 42% of cases (five out of 12 patients) [17,18,19,20,24], whereas heart failure became apparent in 9% (one out of 11 patients) [19] and immunologic phenomena in 8% (one out of 12 patients) [23]. Furthermore, 8% (one patient) had a paravalvular abscess [16] and 8% (one patient) developed a mycotic aneurysm [26].

### 3.7. Treatment and Outcomes of IE by Yersinia Species

Treatment administered for IE by *Yersinia* species can be seen in detail in Supplementary Table S1 and in summary in Table 1. The duration of treatment among survivors ranged from 2 to 16 weeks, with a median duration of 6 weeks [10,17,19,20,21,22,23,25,26]. Surgical management along with antimicrobial use was performed in 25% of patients (three out of 12 patients) [19,20,24]. Clinical cure was achieved in 83% (10 out of 12 patients) and overall mortality was 17% (two patients) and was attributed directly to IE [10,16,17,18,19,20,21,22,23,24,25,26].

### 3.8. Statistical Analysis of IE by Yersinia Species

We performed a univariate logistic regression analysis to identify any potential association between overall mortality and the following parameters: gender, age, having rheumatic heart disease, having a prosthetic cardiac valve, positive stool culture, having IE by *Y. enterocolitica* serotype O:3, cephalosporin or aminoglycoside resistance, aortic, mitral or tricuspid valve IE, developing fever, sepsis, heart failure, embolic or immunologic phenomena, developing a paravalvular abscess or mycotic aneurysm, treatment with cephalosporins, aminoglycosides or quinolones and having a surgery in combination with antimicrobial treatment. Based on the aforementioned analysis, a statistically significant positive association of overall mortality with development of paravalvular abscess (*p* = 0.02) was noted. The results of the regression analysis can be seen in Supplementary Table S2.

## 4. Discussion

In this study, we identified *Y. enterocolitica* as the only species causing IE among the *Yersinia* genus. The mitral valve was the most commonly infected site, while diagnosis was facilitated by transthoracic echocardiography in more than half of the cases. The most frequent clinical presentations were fever and sepsis. Aminoglycosides, cephalosporins and quinolones were the most commonly used antimicrobials, while less than 15% of patients died.

*Yersinia* spp., with the exception of *Y. pestis*, which is the cause of plague, are known to cause a variety of infections such as enterocolitis, mesenteric adenitis and urinary, respiratory and musculoskeletal infections, and on rare occasions, they may cause bacteremia or IE [1]. Septicemia by *Y. enterocolitica* mostly affects people with predisposing conditions such as chronic liver disease, alcoholism, malnutrition, immunosuppression or iron overload [1,27,28]. IE is a quite rare complication of *Yersinia* spp. Local abnormalities in the valves may play an important role in the pathophysiology by facilitating the targeting of the valvular tissue by *Yersinia* and subsequent bacterial growth. To that end, three types of adhesins that are produced by enteropathogenic strains of *Yersinia* may be of clinical importance, namely invasin, YadA and Ail [29,30]. Among these adhesins, YadA may be the most important virulent factor and also the most important for the pathophysiology of IE since it allows *Yersinia* to adhere to cells and extracellular matrix, resist phagocytosis and auto-agglutinate [29]. On the other hand, the ability of *Yersinia* spp. to form biofilms implies that attachment to a cardiac valve may allow them to multiply and then generate biofilms, thus leading to IE [31,32].

IE is a rare but potentially lethal disease that is usually derived from Gram-positive microorganisms. However, Gram-negative microorganisms can account for some cases of IE, especially in patients who have been previously exposed to the healthcare system [6,33]. In particular, IE caused by *Yersinia* species comprises a very rare disease that has been mainly analyzed through case reports. To our knowledge, this is the first study that provides a comprehensive review about IE by *Yersinia* species and provides information on its epidemiology, microbiology, treatment and outcomes.

The mean age at diagnosis of patients with *Yersinia* IE was 67 years, which is slightly higher than the age at diagnosis of IE by non-HACEK Gram-negative bacilli in the literature, which is in the range of 40 to 63 years [6,7,8,9,34]. A male predominance among patients with IE by *Yersinia* species was noted, as in other cases of IE caused by non-HACEK Gram-negative bacilli [6,7,8,9,34]. A prosthetic valve was present in 17% of patients with IE by *Yersinia*, which is lower than the rate mentioned in other studies of IE by non-HACEK Gram-negative bacilli, which is between 25% and 59% [6,7,8,9,34].

The most commonly infected intracardiac sites were the mitral valve in 58% of patients and the aortic valve in 33%. The aforementioned data are in complete agreement with a study with IE by non-HACEK Gram-negative bacilli, where the mitral valve was the most commonly infected valve in 31% of patients, followed by the aortic valve in 24% [28]. However, other studies have mentioned that the aortic valve was the most commonly infected valve in 33% to 45% of cases, followed by either the mitral valve in 27% to 40% of patients [9,35] or the tricuspid valve in 33% of patients [29].

Regarding patients’ clinical signs, fever was the most common symptom, occurring in 92% of patients, while 70% of patients were septic. In studies with IE caused by non-HACEK Gram-negative bacilli, presence of fever was noted in 91% to 100% [6,7,8,9] and sepsis in 79% to 85% of patients [8,9]. Notably, 15% of patients with IE caused by *Yersinia* developed heart failure, a proportion similar to that in cases of non-HACEK Gram-negative IE which ranged from 8% to 37% [6,7,8,9]. Embolic phenomena in *Yersinia* IE were present in 42% of patients, which is in line with other cases of IE by non-HACEK Gram-negative bacilli, where the rate is from 17% to 65%, while immunologic phenomena were present in 8% of patients, which is lower than the corresponding rate in non-HACEK Gram-negative bacilli IE, where that rate is between 14% and 27% [6,7,8,9,34]. Among patients with IE caused by *Yersinia*, 8% presented a paravalvular abscess, which is lower than the corresponding rate in IE by non-HACEK Gram-negative bacilli, which was in the range of 13% to 42% [6,7,8,9]. Interestingly, in our study, presence of a paravalvular abscess was associated with increased mortality.

Regarding antimicrobial resistance, *Y. enterocolitica* has varying antimicrobial susceptibility patterns among serogroups but is generally considered susceptible in vitro to aminoglycosides, third-generation cephalosporins, quinolones, co-trimoxazole, tetracycline and chloramphenicol, but resistant to penicillin, aminopenicillins and first-generation cephalosporins, due to the chromosomally encoded beta-lactamase-producing genes blaA and blaB [2,35]. Herein, we confirmed that *Y. enterocolitica* presents high resistance to aminopenicillins, but we also noted alarming rates of resistance to quinolones, which are considered the first-line treatment in infections by this pathogen [2]. This is in contrast to another study that shows minimal resistance rates to quinolones [36]. Importantly, resistance to cephalosporins, another class of antimicrobials commonly used in the treatment of these infections, was 18%, while resistance to aminoglycosides, an antimicrobial commonly used for synergy in serious infections and IE, was less than 10%.

As expected, aminoglycosides, cephalosporins and quinolones were used in 83%, 42% and 42% of cases, respectively. These rates may have been the highest among the antimicrobials used, but they should be read with caution, since in some older studies in this systematic review, quinolones were not available. With the above-mentioned data, it is reasonable to suggest that treatment of IE by *Yersinia* spp. should include a combination of a third-generation cephalosporin or a quinolone with an aminoglycoside. However, a preference towards cephalosporins over quinolones in the case of IE by non-HACEK Gram-negative bacteria is noted in the guidelines [37]. In all instances, treatment should be guided based on the results of antimicrobial susceptibility testing. Duration of treatment should be at least 6 weeks, as suggested by the guidelines [37].

Mortality was relatively low, with about 17% of patients dying, all due to IE. This rate was relatively lower than the one in studies of IE by non-HACEK Gram-negative bacilli, where mortality was as high as 44% [6,7,8,9,34].

This systematic review has some limitations that should be acknowledged. First and foremost, the number of studies in the literature is very small and stems only from case reports due to the rarity of this disease; thus, the results should be interpreted cautiously, since the quality of evidence contributed by these studies was low to very low. On the other hand, the possibility of publication bias also exists. However, since no study giving information specifically on *Yersinia* IE with an adequate number of patients could be found in the literature, this is the only methodology that could have been used in order to study these infections.

## 5. Conclusions

This systematic review describes the epidemiology, clinical characteristics, microbiology, treatment and outcomes of IE caused by *Yersinia* species. *Y. enterocolitica* was the only identified species, while alarming resistance rates were noted for commonly used antimicrobials. Presence of a paravalvular abscess was associated with increased mortality.

## Figures and Tables

**Table 1:** Characteristics of 12 patients with infective endocarditis by *Yersinia* species. Values show cases among patients with available data.

Characteristic	Value
Male, *n* (%)	10/12 (83%)
Age, mean (SD) in years	67 (13)
Predisposing factors	
Prosthetic valve, *n* (%)	2/12 (17%)
Rheumatic heart disease, *n* (%)	3/12 (25%)
Site of infection inside heart	
Mitral valve, *n* (%)	7/12 (58%)
Aortic valve, *n* (%)	4/12 (33%)
Tricuspid valve, *n* (%)	2/12 (17%)
Multiple valves, *n* (%)	1/12 (8%)
Antimicrobial resistance	
Aminopenicillins, *n* (%)	5/7 (71%)
Quinolones, *n* (%)	2/8 (25%)
Chloramphenicol, *n* (%)	1/5 (20%)
Cephalosporins, *n* (%)	2/11 (18%)
Co-trimoxazole, *n* (%)	1/6 (17%)
Tetracycline, *n* (%)	1/6 (17%)
Carbapenems, *n* (%)	1/7 (13%)
Aminoglycosides, *n* (%)	1/12 (8%)
Method of anatomical diagnosis	
Transthoracic echocardiography, *n* (%)	7/12 (58%)
Transesophageal echocardiography, *n* (%)	4/12 (33%)
Autopsy, *n* (%)	1/12 (8%)
Clinical characteristics	
Fever, *n* (%)	11/12 (92%)
Septic, *n* (%)	7/10 (70%)
Embolic phenomena, *n* (%)	5/12 (42%)
Heart failure, *n* (%)	1/11 (9%)
Immunologic phenomena, *n* (%)	1/12 (8%)
Mycotic aneurysm, *n* (%)	1/12 (8%)
Paravalvular abscess, *n* (%)	1/12 (8%)
Treatment	
Duration of treatment in weeks, median (IQR)	6 (4.5–10)
Aminoglycosides, *n* (%)	10/12 (83%)
Cephalosporin, *n* (%)	5/12 (42%)
Quinolone, *n* (%)	5/12 (42%)
Aminopenicillin, *n* (%)	2/12 (17%)
Co-trimoxazole, *n* (%)	2/12 (17%)
Surgical management, *n* (%)	3/12 (25%)
Outcomes	
Clinical cure, *n* (%)	10/12 (83%)
Deaths due to infection, *n* (%)	2/12 (17%)
Deaths overall, *n* (%)	2/12 (17%)

IQR: interquartile range; SD: standard deviation.

## Data Availability

The datasets analyzed during the current study are available from the corresponding author upon reasonable request.

## Supplementary Materials

The following are available online at https://www.mdpi.com/2414-6366/6/1/19/s1, Table S1: Characteristics of the included studies. Table S2. Univariate analysis of mortality in patients with IE by Yersinia species.

## References

[1] Bennett J.E., Dolin E., Blaser M.J. (2019). Mandell, Douglas, And Bennett’s Principles and Practice of Infectious Diseases.

[2] Fàbrega A., Vila J. (2012). *Yersinia enterocolitica*: Pathogenesis, virulence and antimicrobial resistance. Enferm. Infecc. Microbiol. Clin..

[3] Asplund K., Johansson T., Siitonen A. (1998). Evaluation of pulsed-field gel electrophoresis of genomic restriction fragments in the discrimination of *Yersinia enterocolitica* O:3. Epidemiol. Infect..

[4] Wang A., Gaca J.G., Chu V.H. (2018). Management Considerations in Infective Endocarditis: A Review. JAMA.

[5] Baddour L.M., Wilson W.R., Bayer A.S., Fowler V.G., Tleyjeh I.M., Rybak M.J., Barsic B., Lockhart P.B., Gewitz M.H., Levison M.E. (2015). Infective Endocarditis in Adults: Diagnosis, Antimicrobial Therapy, and Management of Complications: A Scientific Statement for Healthcare Professionals from the American Heart Association. Circulation.

[6] Morpeth S., Murdoch D., Cabell C.H., Karchmer A.W., Pappas P., Levine D., Nacinovich F., Tattevin P., Fernández-Hidalgo N., Dickerman S. (2007). Non-HACEK Gram-negative bacillus endocarditis. Ann. Intern. Med..

[7] Loubet P., Lescure F.X., Lepage L., Kirsch M., Armand-Lefevre L., Bouadma L., Lariven S., Duval X., Yazdanpanah Y., Joly V. (2015). Endocarditis due to Gram-negative bacilli at a French teaching hospital over a 6-year period: Clinical characteristics and outcome. Infect. Dis. (Lond).

[8] Ioannou P., Mavrikaki V., Kofteridis D.P. (2020). Infective endocarditis by *Acinetobacter* species: A systematic review. J. Chemother..

[9] Ioannou P., Vougiouklakis G. (2020). Infective endocarditis by *Proteus* species: A systematic review. Germs.

[10] Lupi A., Poletti F., Mondino V., Canale C., Leonardo L., Rognoni A., Sante Bongo A., Caimmi P.P., Nardi F. (2013). Subacute endocarditis caused by *Yersinia enterocolitica*: A case report. Scand. J. Infect. Dis..

[11] Stroup D.F., Berlin J.A., Morton S.C., Olkin I., Williamson G.D., Rennie D., Olkin I., Williamson G.D., Rennie D., Moher D. (2000). Meta-analysis of observational studies in epidemiology: A proposal for reporting. Meta-analysis Of Observational Studies in Epidemiology (MOOSE) group. JAMA.

[12] Wallace B.C., Small K., Brodley C.E., Lau J., Trikalinos T.A. (2012). Deploying an interactive machine learning system in an evidence-based practice center: Abstrackr. Proc. ACM. Int. Health Inform. Symp. IHI.

[13] Li J.S., Sexton D.J., Mick N., Nettles R., Fowler V.G., Ryan T., Bashore T., Corey G.R. (2000). Proposed modifications to the Duke criteria for the diagnosis of infective endocarditis. Clin. Infect. Dis..

[14] Bone R.C., Balk R.A., Cerra F.B., Dellinger R.P., Fein A.M., Knaus W.A., Schein R.M., Sibbald W.J. (1992). Definitions for sepsis and organ failure and guidelines for the use of innovative therapies in sepsis. The ACCP/SCCM Consensus Conference Committee. American College of Chest Physicians/Society of Critical Care Medicine. Chest.

[15] Guyatt G.H., Oxman A.D., Vist G.E., Kunz R., Falck-Ytter Y., Alonso-Coello P., Schünemann H.J., GRADE Working Group (2008). GRADE: An emerging consensus on rating quality of evidence and strength of recommendations. BMJ.

[16] Urbano-Márquez A., Estruch R., Agustí A., Jimenez De Anta M.T., Ribalta T., Grau J.M., Rozman C. (1983). Infectious endocarditis due to *Yersinia enterocolitica*. J. Infect. Dis..

[17] Appelbaum J.S., Wilding G., Morse L.J. (1983). *Yersinia enterocolitica* endocarditis. Arch. Intern. Med..

[18] Green H.T., Morris A.I., Haqqani M.T., Nair P. (1983). Infective endocarditis due to *Yersinia enterocolitica*. J. Infect..

[19] Foberg U., Frydén A., Kihlström E., Persson K., Weiland O. (1986). *Yersinia enterocolitica* septicemia: Clinical and microbiological aspects. Scand. J. Infect. Dis..

[20] Giamarellou H., Antoniadou A., Kanavos K., Papaioannou C., Kanatakis S., Papadaki K. (1995). *Yersinia enterocolitica* endocarditis: Case report and literature review. Eur. J. Clin. Microbiol. Infect. Dis..

[21] Bonnet E., Archambaud M., Sommabere A., Suc C., Elias Z., Gallinier M., Massabuau P., Bounhoure J.P., Massip P. (1998). Endocarditis due to *Yersinia enterocolitica*. Infection.

[22] Le Moal G., Roblot F., Paccalin M., Breux J.P., Becq-Giraudon B. (2001). Pacemaker endocarditis due to *Yersinia enterocolitica*. Scand. J. Infect. Dis..

[23] Karachalios G., Bablekos G., Karachaliou G., Charalabopoulos A.K., Charalabopoulos K. (2002). Infectious endocarditis due to *Yersinia enterocolitica*. Chemotherapy.

[24] Papaioannou C.A., Varvarigos N., Karatsolis G., Papaioannou N., Draganigos A., Katsantouris C., Kappas A., Avramopoulou T. (2003). *Yersinia Enterocolitica* Endocarditis. Hell. J. Cardiol..

[25] Krajinović V., Tambić Andrasević A., Barsić B. (2007). Tricuspidal valve endocarditis due to *Yersinia enterocolitica*. Infection.

[26] Mason J., Lal P., Torella F., Sharma A., Cooke R., Anson J. (2014). *Yersinia enterocolitica*: A Rare Cause of Infective Endocarditis and Mycotic Aneurysm. Clin. Microbiol. Newsl..

[27] Lenz T., Schulte K.L., Meyer-Sabellek W. (1984). *Yersinia enterocolitica* septicemia during long-term immunosuppressive treatment. J. Infect. Dis..

[28] Piroth L., Meyer P., Bielefeld P., Besancenot J.F. (1997). *Yersinia* bacteremia and iron overload. Rev. Med. Interne..

[29] Leo J.C., Skurnik M. (2011). Adhesins of human pathogens from the genus *Yersinia*. Adv. Exp. Med. Biol..

[30] Emödy L., Heesemann J., Wolf-Watz H., Skurnik M., Kapperud G., O’Toole P., Wadström T. (1989). Binding to collagen by *Yersinia enterocolitica* and *Yersinia pseudotuberculosis*: Evidence for yopA-mediated and chromosomally encoded mechanisms. J. Bacteriol..

[31] Vestby L.K., Grønseth T., Simm R., Nesse L.L. (2020). Bacterial Biofilm and its Role in the Pathogenesis of Disease. Antibiotics.

[32] Lenchenko E., Lozovoy D., Strizhakov A., Vatnikov Y., Byakhova V., Kulikov E., Sturov N., Kuznetsov V., Avdotin V., Grishin V. (2019). Features of formation of *Yersinia enterocolitica* biofilms. Vet. World.

[33] Cahill T.J., Prendergast B.D. (2016). Infective endocarditis. Lancet.

[34] Veve M.P., McCurry E.D., Cooksey G.E., Shorman M.A. (2020). Epidemiology and outcomes of non-HACEK infective endocarditis in the southeast United States. PLoS ONE.

[35] Bent Z.W., Young G.M. (2010). Contribution of BlaA and BlaB beta-lactamases to antibiotic susceptibility of *Yersinia enterocolitica* biovar 1B. Antimicrob. Agents. Chemother.

[36] Frazão M.R., Andrade L.N., Darini A.L.C., Falcão J.P. (2017). Antimicrobial resistance and plasmid replicons in *Yersinia enterocolitica* strains isolated in Brazil in 30 years. Braz. J. Infect. Dis..

[37] Habib G., Lancellotti P., Antunes M.J., Bongiorni M.G., Casalta J.P., Del Zotti F., Dulgheru R., El Khoury G., Erba P.A., Iung B. (2015). 2015 ESC Guidelines for the management of infective endocarditis: The Task Force for the Management of Infective Endocarditis of the European Society of Cardiology (ESC). Endorsed by: European Association for Cardio-Thoracic Surgery (EACTS), the European Association of Nuclear Medicine (EANM). Eur. Heart. J..

