# Supplementary material for: Infective Endocarditis by Yersinia Species: A Systematic Review
Source: Trop Med Infect Dis. 2021 Feb 2;6(1):19. doi: 10.3390/tropicalmed6010019 (PMC7931003; doi:10.3390/tropicalmed6010019)
Supplement: Supplementary file 1 [file tropicalmed-06-00019-s001.zip › Supplementary Table 2.docx]

**Supplementary Table 2.** Univariate analysis of mortality in patients with IE by *Yersinia* species.

| Parameter in univariate analysis | p |
| --- | --- |
| Gender | 0.5331 |
| Age | 0.7454 |
| Rheumatic heart disease | 0.4178 |
| Prosthetic cardiac valve | 0.5331 |
| Positive stool culture | 0.1976 |
| IE by Y. enterocolitica serotype O:3 | 0.0769 |
| Cephalosporin resistance | 0.6618 |
| Aminoglycoside resistance | 0.6761 |
| Aortic valve IE | 0.3166 |
| Mitral valve IE | 0.2258 |
| Tricuspid valve IE | 0.5331 |
| Fever | 0.6761 |
| Sepsis | 0.3559 |
| Heart failure | 0.7699 |
| Embolic phenomena | 0.8154 |
| Paravalvular abscess | 0.0162 |
| Mycotic aneurysm | 0.6761 |
| Treatment with cephalosporins | 0.2258 |
| Treatment with aminoglycosides | 0.5331 |
| Treatment with quinolones | 0.2258 |
| Surgery along with antimicrobial treatment | 0.4178 |
