# Supplementary material for: Infective Endocarditis by Yersinia Species: A Systematic Review
Source: Trop Med Infect Dis. 2021 Feb 2;6(1):19. doi: 10.3390/tropicalmed6010019 (PMC7931003; doi:10.3390/tropicalmed6010019)
Supplement: Supplementary file 1 [file tropicalmed-06-00019-s001.zip › Supplementary Table 1.docx]

**Table 1.** Characteristics of the included studies.

| **Study, year published** | **Number of patients** | **Age (years)** | **Gender** | **Site of infection, n (%)** | **Microbiology of infection, n (%)** | **Treatment administered** | **Infection Outcomes, n (%)** |
| --- | --- | --- | --- | --- | --- | --- | --- |
| Urbano-Márquez et al, 1983 [16] | 1 | 72 | Male | MV 1 (100) | *Y. enterocolitica* 1 (100) | Aminopenicillin 1 (100)  Aminoglycoside 1 (100) | Clinical cure^a^ 0 (0)  Overall mortality 100 (100)  IE specific mortality 1 (100) |
| Appelbaum et al, 1983 [17] | 1 | 73 | Female | AoV 1 (100) | *Y. enterocolitica* 1 (100) | Aminoglycoside 1 (100) | Clinical cure 1 (100)  Overall mortality 0 (0) |
| Green et al, 1983 [18] | 1 | 68 | Male | MV 1 (100) | *Y. enterocolitica* 1 (100) | Aminoglycoside 1 (100)  Co-trimoxazole 1 (100) | Clinical cure 0 (0)  Overall mortality 100 (100)  IE specific mortality 1 (100) |
| Foberg et al, 1986 [19] | 1 | 61 | Male | AoV 1 (100) | *Y. enterocolitica* 1 (100) | Co-trimoxazole 1 (100)  Aminoglycoside 1 (100)  Cephalosporin 1 (100)  Surgical replacement 1 (100) | Clinical cure 1 (100) |
| Giamarellou et al, 1995 [20] | 1 | 45 | Male | Prosthetic MV 1 (100) | *Y. enterocolitica* 1 (100) | Cephalosporin 1 (100)  Aminoglycoside 1 (100)  Surgical replacement 1 (100) | Clinical cure 1 (100) |
| Bonnet et al, 1998 [21] | 1 | 68 | Male | MV 1 (100) | *Y. enterocolitica* 1 (100) | Quinolone 1 (100)  Aminoglycoside 1 (100) | Clinical cure 1 (100) |
| Le Moal et al, 2001 [22] | 1 | 89 | Male | TrV 1 (100) | *Y. enterocolitica* 1 (100) | Cephalosporin 1 (100)  Quinolone 1 (100)  Pacemaker removal 1 (100) | Clinical cure 1 (100) |
| Karachalios et al, 2002 [23] | 1 | 58 | Male | MV 1 (100) | *Y. enterocolitica* 1 (100) | Quinolone 1 (100)  Aminoglycoside 1 (100) | Clinical cure 1 (100) |
| Papaioannou et al, 2003 [24] | 1 | 45 | Male | Prosthetic MV 1 (100) | *Y. enterocolitica* 1 (100) | Cephalosporin 1 (100)  Aminoglycoside 1 (100)  Surgical replacement 1 (100) | Clinical cure 1 (100) |
| Krajinović et al, 2007 [25] | 1 | 75 | Male | TrV 1 (100) | *Y. enterocolitica* 1 (100) | Quinolone 1 (100)  Aminopenicillin 1 (100)  Aminoglycoside 1 (100) | Clinical cure 1 (100) |
| Lupi et al, 2013 [10] | 1 | 73 | Female | AoV 1 (100) | *Y. enterocolitica* 1 (100) | Cephalosporin 1 (100)  Aminoglycoside 1 (100) | Clinical cure 1 (100) |
| Mason et al, 2014 [26] | 1 | 78 | Male | AoV 1 (100)  MV 1 (100) | *Y. enterocolitica* 1 (100) | Quinolone 1 (100) | Clinical cure 1 (100) |

^a^ Defined as clinical resolution of the infection as a result of treatment.

AoV: aortic valve; LV: left ventricle; MV: mitral valve, NR: not reported; TrV: tricuspid valve;
